# Supplementary material for: Anatomical and physiological foundations of cerebello-hippocampal interaction
Source: eLife. 2019 Jun 17;8:e41896. doi: 10.7554/eLife.41896 (PMC6579515; doi:10.7554/eLife.41896)
Supplement: Transparent reporting form [file elife-41896-transrepform.docx]

***eLife’s* transparent reporting form**

We encourage authors to provide detailed information *within their submission* to facilitate the interpretation and replication of experiments. Authors can upload supporting documentation to indicate the use of appropriate reporting guidelines for health-related research (see [EQUATOR Network](http://www.equator-network.org/%20)), life science research (see the [BioSharing Information Resource](https://biosharing.org/)), or the [ARRIVE guidelines](http://www.plosbiology.org/article/info:doi/10.1371/journal.pbio.1000412) for reporting work involving animal research. Where applicable, authors should refer to any relevant reporting standards documents in this form.

If you have any questions, please consult our Journal Policies and/or contact us: [editorial@elifesciences.org](mailto:editorial@elifesciences.org).

**Sample-size estimation**

- You should state whether an appropriate sample size was computed when the study was being designed
- You should state the statistical method of sample size computation and any required assumptions
- If no explicit power analysis was used, you should describe how you decided what sample (replicate) size (number) to use

Please outline where this information can be found within the submission (e.g., sections or figure legends), or explain why this information doesn’t apply to your submission:

No explicit power analysis was used. We indicate all sample sizes in the figure legends and the methods section (lines 624-626).

The sample size used for the anatomical tracing study was similar to that found in similar studies in the literature. For the electrophysiology study the initial sample size was set to have enough individuals reaching the final stages of training (goal directed behavior in a virtual environment).

**Replicates**

- You should report how often each experiment was performed
- You should include a definition of biological versus technical replication
- The data obtained should be provided and sufficient information should be provided to indicate the number of independent biological and/or technical replicates
- If you encountered any outliers, you should describe how these were handled
- Criteria for exclusion/inclusion of data should be clearly stated
- High-throughput sequence data should be uploaded before submission, with a private link for reviewers provided (these are available from both GEO and ArrayExpress)

Please outline where this information can be found within the submission (e.g., sections or figure legends), or explain why this information doesn’t apply to your submission:

The number of animals (anatomy) or animals/recordings (electrophysiology) used for the analysis is available in the figure legends and the method sections.

For the anatomical tracing study the inclusion of the data was determined after the injection site and spreading verification. For the electrophysiology, the recordings from off-target electrodes were excluded from all analysis.

**Statistical reporting**

- Statistical analysis methods should be described and justified
- Raw data should be presented in figures whenever informative to do so (typically when N per group is less than 10)
- For each experiment, you should identify the statistical tests used, exact values of N, definitions of center, methods of multiple test correction, and dispersion and precision measures (e.g., mean, median, SD, SEM, confidence intervals; and, for the major substantive results, a measure of effect size (e.g., Pearson's r, Cohen's d)
- Report exact p-values wherever possible alongside the summary statistics and 95% confidence intervals. These should be reported for all key questions and not only when the p-value is less than 0.05.

Please outline where this information can be found within the submission (e.g., sections or figure legends), or explain why this information doesn’t apply to your submission:

The specific statistical analysis are indicated in every figure legend and results section. As indicated in the “Statistical analysis” section of methods (lines 839-842), normality was assessed using a Shapiro-Wilk test and parametric or non-parametric test were used accordingly. When one of the compared groups was non-normal, non-parametric test were used.

(For large datasets, or papers with a very large number of statistical tests, you may upload a single table file with tests, Ns, etc., with reference to sections in the manuscript.)

**Group allocation**

- Indicate how samples were allocated into experimental groups (in the case of clinical studies, please specify allocation to treatment method); if randomization was used, please also state if restricted randomization was applied
- Indicate if masking was used during group allocation, data collection and/or data analysis

Please outline where this information can be found within the submission (e.g., sections or figure legends), or explain why this information doesn’t apply to your submission:

For the anatomical tracing the groups are established by post-injection survival time in as indicated in the results section (lines 98-99), the figures 1, S1, S2 and S3 legends and the method section (lines 647-648).

For the electrophysiology, analysis groups were established after completion of all experiments and histological verification of electrodes placement as indicated in the results section (lines 219-220).

**Additional data files (“source data”)**

- We encourage you to upload relevant additional data files, such as numerical data that are represented as a graph in a figure, or as a summary table
- Where provided, these should be in the most useful format, and they can be uploaded as “Source data” files linked to a main figure or table
- Include model definition files including the full list of parameters used
- Include code used for data analysis (e.g., R, MatLab)
- Avoid stating that data files are “available upon request”

Please indicate the figures or tables for which source data files have been provided:

Individual source data are not provided in form of numerical data or summary table as most of the individual values are plotted in all the graphs and the mean and SEM are also plotted or indicated at the legends.

The Matlab codes used for the spectral analysis are part of a fully available, well documented toolbox (Chronux toolbox) and referenced in the text. The parameters used for these codes is indicated in method section lines 818-822.
